# Patterns in Abundance and Seasonality of Insects in the Siruvani Forest of Western Ghats, Nilgiri Biosphere Reserve, Southern India

**DOI:** 10.1100/tsw.2004.33

**Published:** 2004-06-08

**Authors:** P. R. Arun, V. S. Vijayan

**Affiliations:** Sálim Ali Centre for Ornithology and Natural History, Coimbatore, India 641 108, India

**Keywords:** India, insect seasonality, net sweep method, seasonal pattern, Western Ghats

## Abstract

The seasonal abundance patterns of insects inhabiting the understory vegetation of a mixed deciduous forest were examined with the help of the sweep-net sampling method. During the study period of 2 years, insects were sampled regularly from the understory vegetation of the three selected habitats (moist-deciduous, riverine, and teak plantation) of the mixed deciduous forest. Insect abundance was maximum in the moist-deciduous habitat and minimum in the teak plantation. Generally, insect abundance was the highest during the southwest monsoon in all habitats. The temporal pattern of fluctuations in the insect abundance followed more or less the same pattern in all the three habitats studied. The insect abundance of the understory vegetation varied among the habitats studied, while the pattern of seasonal fluctuations in insect abundance was comparable among habitats. Composition of the insect community also indicated prominent seasonal changes within habitats than interhabitat changes within a season.

## INTRODUCTION

Seasonal variation in the abundance of a species is an adaptive phenomenon evolved to take maximum advantage from the ambient environmental conditions. Presence of distinct larval and imago stages, which differ from each other in many ways, including the feeding mechanisms and even the ecological niche, is a characteristic feature of holometabolous insects. Equipped with the diverse modes of lifestyles and ecological requirements during different life stages, the insects have got an edge over most other organisms in resource utilization potential. One of the major factors on which their success and survival depends is how efficiently they time their lifecycle so as to utilize the maximum resources and environmental conditions. Apart from the primary genetic programming, environmental conditions that involve both density-dependent as well as density-independent factors also play an important part in insect seasonality. Density-dependent factors are responsible for regulating the population about its average level of abundance, while the density-independent factors, such as climate and weather, can cause catastrophic changes in the abundance either by enhancing the mortality rate or by triggering a change in the neurosecretory cells (known as “endocrine switch”) of the brain, which in turn switch on or off the various adaptive physiological mechanisms involved in the survival strategy, such as diapause and pupation.

In the present study, the pattern of fluctuations in the overall insect abundance was found less predominant compared to that in its component groups, such as orders, families, and species. This was because of the difference in the pattern of abundance of different component groups in the total abundance. For instance, the Diptera were highly abundant in August, while Lepidoptera and Orthoptera showed low abundance during this month, while an opposite trend was observed in December. The relative fluctuations in the insect abundance showed more definite patterns in the case of smaller and less diverse groups, compared to larger and more complex taxonomic groups. Hence, the fluctuation in abundance at the family level tends to be more prominent than that at the order level. Among the environmental parameters, temperature was the important factor in the seasonal fluctuations of Diptera, while abundance of Orthoptera was related with bird abundance[1]. In the case of butterflies, the flower abundance was a key factor that affected the abundance, especially in the families Satyridae, Papilionidae, and Hesperiidae[1,2]. The present paper examines any direct relation between the fluctuations in insect abundance with abiotic environmental parameters such as rainfall, temperature, and humidity. A direct linear relationship between the abundance and environmental factors was absent in most of the insect orders, but a combination of factor appears to explain the abundance fluctuations better. However, for a thorough understanding of the mechanisms and factors involved in the seasonality, species-specific studies on long-term basis have to be conducted.

Natural insect communities are vastly diverse with highly complex ecological interactions. Depicting a direct “cause and effect relationship” with normal environmental variables in such communities would be highly difficult. The amount of present knowledge on the insects of the tropical forests is comparatively sparse compared to that of the temperate forests. Although the insects form around 50% of the biota described from India[3], little is known of their ecology and seasonal abundance patterns in the natural ecosystems. Generally, the most studied group of insects is that of agricultural pests, which forms only a minuscule part of the enormously diverse insect population[4]. The lack of scientific tradition and moderate budgets are the two major causes attributed to this lack of knowledge[5]. Except for a few recent studies[2,6], information on seasonality of insects is grossly lacking from the Western Ghats region, one of the two internationally recognized biodiversity hotspots of the Indian region. However, accounts of the seasonal abundance of insects are available in many of the ecological studies, especially the ornithological studies reported from various parts of India[7,8,9,10,11,12]. These studies attempted to establish the relationship between the breeding seasons of birds and the insect seasonality. The present study was aimed at elucidating the temporal pattern in the abundance of insects and the environmental mechanism responsible for bringing about this pattern.

The temporal fluctuations in the abundance are an important manifestation of populations’ response to the environmental conditions. Such seasonal fluctuations are especially prominent in the populations of lower organisms such as insects[13,14,15,16,17]. Insects are generally more sensitive to environmental changes and the various distinct life-history stages of insects are adaptively timed according to the seasonal environmental conditions[18]. This adaptive seasonal timing is an important reason for the evolutionary success of insects, which helped them to persist so long in geological time since the Carboniferous period of the Palaeozoic era. In the present study, the seasonal abundance patterns of different insect orders from a moist-deciduous forest of the Western Ghats region of South India were examined with reference to the interhabitat differences in seasonality and abundance. The 2-year study was carried out between September 1994 to August 1996.

## STUDY AREA

The selected study area (76$^o$41, 45' E and 10$^o$56, 59' N) is about 35 km west of the city of Coimbatore in Tamilnadu and is situated at the foothills of the eastern slope of the Western Ghats, one among the biodiversity hotspots of the world (Fig. 1).

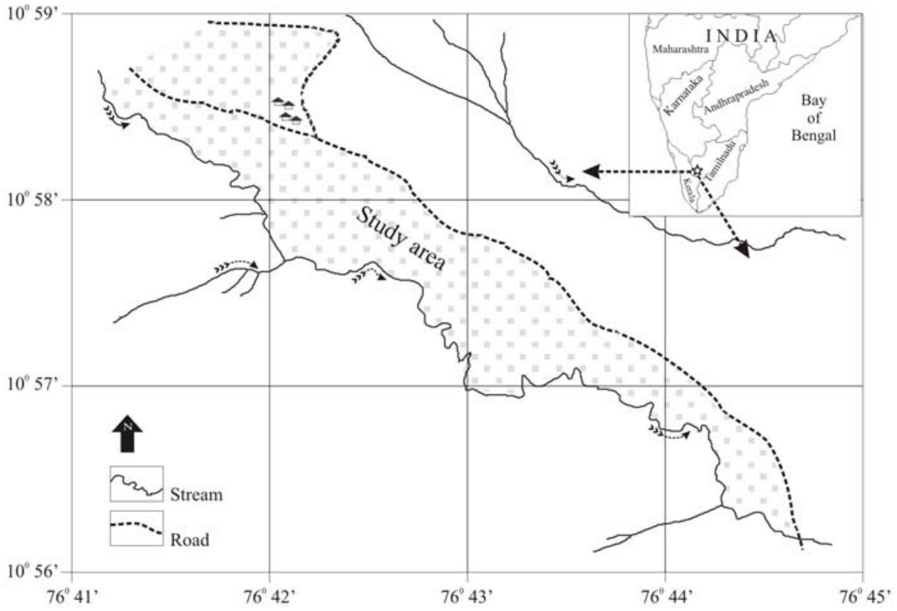

**FIGURE 1.** Map of the study area.

The study area had seen high human disturbance especially from cattle grazing. The road that connects the forest check post and the water treatment plant of the Tamilnadu Water supply and Drainage (TWAD) Board, around 6 km inside the forest, is the main access road for the study area and it also borders the study area along its northeastern aspect for around 2.5 km. The area is contiguous with one of the core area evergreen forests of the Nilgiri Biosphere Reserve, in the Western Ghats mountain range of the Indian peninsula. Being situated within the Western Ghats mountain range, it supports a vast and diverse flora and fauna. Human habitations such as the staff quarters of TWAD and the Coimbatore Municipal Corporation were close to the study area. Two major tribal settlements of the area (Vellapathy and Sarkaraporathy) were also in close proximity. Since the study area was located close to the forest edge, there were various anthropogenic pressures. Around 100 cattle regularly grazed inside the forest, including the study area. Invasive and alien weedy plant species such as *Lantana camara* and *Chromolaena odorata* were abundantly present in the area. *L. camara* formed the dominant understory vegetation in most of the areas.

## METHODS

Sampling of insects was done using a sweep net following standard methods[5,19,20]. The sweep samplings were done from the undergrowth (herb and shrub layers), while walking along a transect route of about 1.5 km. The net used for the sweeping had a circular mouthed frame with a diameter of 30 cm, which was fitted with a conical cotton net 75-cm long from mouth to tip of the tail. All the sweeps were made by the same person using the net of the same dimensions throughout the study period. One hundred sweeps were regarded as one sample and one such sample was taken from each of the three habitats during a sampling cycle. Three such sampling cycles were carried out in each month at an average interval of around 10 days between the cycles. Each sampling cycle covering all the habitats was completed in the same day in all the cases. Habitats were sampled in random order to avoid any possible bias towards a particular habitat due to the difference in the time of sweeping. This was achieved by selecting the habitat to be swept first, second, and third in a day using lottery method.

All the samples were taken between 08.30 and 11.30 AM. The sweep sampling was done from the ground-level vegetation comprised of herbs and shrubs. The insects collected were temporarily immobilized using chloroform vapors and were released back after being sorted out up to order and recorded. No effort was made to identify the insects up to species level. However, the number of morphologically identifiable taxonomic units (MTUs) of insects present in each sample was recorded and was considered as an index of species richness in the present study.

Environmental factors were also monitored regularly. The daily rainfall data were collected from the rain gauge maintained inside the study area by TWAD. Temperature was recorded using a Maximum-minimum thermometer and humidity using a Hair-hygrometer.

## HABITATS

Three habitats (moist-deciduous forest, riverine forest, and teak plantation) within the mixed deciduous forest of Siruvani were selected for the present study. All three habitats were in close proximity within the selected study area and the three transects used for insect sampling in different habitats were only less than a kilometer apart from each other.

The moist-deciduous forest was the major habitat type present in the study area. The shrub layer of this habitat was dominated by the exotic weeds *L. camara* and *Justicia betonica*. The understory vegetation was highly diverse in this habitat compared to other habitats. Dominant tree species were *Grewia tilifolia* and *Terminalia bellarica*.

The riverine habitat was a narrow strip of natural vegetation along the sides of the perennial stream that flows along the southern border of the study site (Fig. 1). Dominant tree species were *Terminalia paniculata* and *T. tomentosa*. Owing to the water availability all through the year, some evergreen species were also present along with the deciduous species. Canopy cover was the highest in this habitat.

The teak plantation included in the study was in an abandoned condition ever since it was planted in 1970s. This man-made habitat was, however, contiguous with the natural moist-deciduous and riverine forests, and was present in the form of an elongated patch sandwiched between them. The dominant species of the understory vegetation in this habitat was *Glycosmis pentaphylla*. Canopy cover and the diversity of understory vegetation were the least in this habitat. Apart from teak (*Tectona grandis*), certain other tree species such as *Terminalia paniculata, Cassia fistula*, and *Butea monosperma* were also present at places.

## SEASONS

The four major seasons discernible in the climate of the region were southwest monsoon (June–August), northeast monsoon (September–November), winter (December–February), and summer (March–May). Southwest monsoon is the major monsoon, during which the area received most of its annual rainfall.

## RESULTS AND DISCUSSION

### Spatio-Temporal Patterns in Insect Abundance

The relative abundance of insects varied among the habitats. Moist-deciduous and riverine forests together accounted for more than 75% of the insect abundance in the area (Fig. 2). The habitat-wise data showed a similar pattern of seasonal fluctuation in the insect abundance in all the three habitats (Fig. 3). Invariably in all the habitats, the southwest monsoon was the most preferred season followed by winter, northeast monsoon, and summer. Table 1 shows the seasonal changes in the composition of the general insect population in the three different habitats (mean number of insects obtained per 100 sweeps is given for each season). Although the composition varied over seasons and habitats, variation between seasons in a particular habitat was more prominent compared to that between habitats in a particular season (Table 1, Fig. 4).

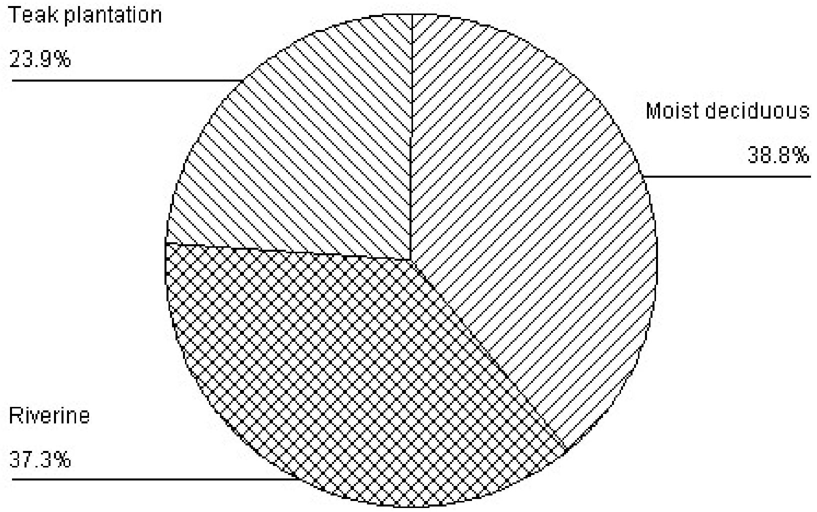

**FIGURE 2**. Spatial pattern of insect abundance.

## Variation in Seasonal Abundance

The relative abundance of insects showed much temporal fluctuation during the 2-year study in all the microhabitats (Fig. 2). The moist-deciduous habitat showed the maximum amount of temporal variation with a covariance of 45.5% followed by the teak plantation (38.29%) and riverine (34.34%) habitats. The least amount of temporal variation in the insect abundance in the riverine habitat might be because of the relatively less seasonal changes in the vegetation diversity and microclimate of the area.

Hierarchical cluster analysis was done using the average linkage between the composition of the insect samples of the three different habitats in four different seasons using correlation measure in order to compare the composition of insect samples. The resulting dendrogram showed higher similarity between samples of the same season especially within wet seasons (southwest and northeast monsoons) and dry season (winter and summer) samples. The insect community composition during the northeast monsoon in moist-deciduous and riverine habitats was more similar to dry season samples, while winter samples from moist-deciduous and riverine habitats formed a separate cluster which was only distantly similar to the dry season samples. The maximum similarity was between the samples of northeast monsoon from the moist-deciduous and teak plantation, and between summer samples from moist-deciduous and teak plantation (Fig. 5).

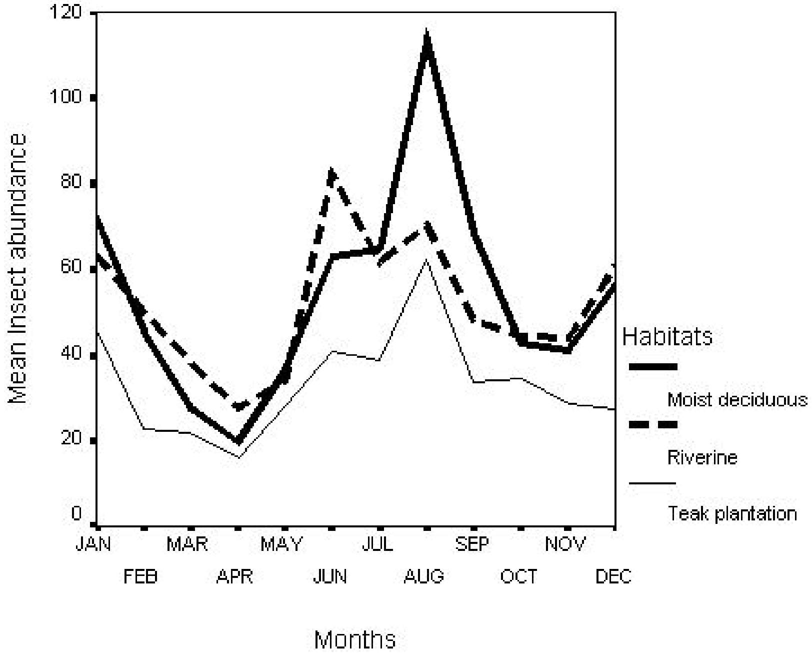

**FIGURE 3.** Pattern of fluctuation in the insect abundance in different habitats.

Although the insect abundance showed a similar trend of fluctuation in all the three habitats, there was significant difference in the insect abundance among these three different habitats at any particular season. Generally, the moist-deciduous forest was the most preferred habitat with maximum abundance of insects, closely followed by the riverine habitat (Fig. 2). The teak plantation was the least preferred habitat with comparatively very low insect abundance during all the seasons.

The abundance of overall insect populations, as well as that of various insect groups, varied among the three habitats. There was significant positive correlation ($p < 0.01$) among the seasonal fluctuations in general insect abundance in all the three habitats studied (Table 2). This significant interhabitat correlation was observed in most of the major insect orders as well.

## Composition of Insect Community

Although there was an apparent difference in insect abundance among the three habitats with moist-deciduous and riverine habitats together accounting for more than 76% of the total insect abundance, the proportion of insect groups in the samples from different habitats did not show much difference except for the very high proportion of Diptera in the moist-deciduous habitat, which was mainly because of the very high abundance of dipterans in that habitat during the first wet season. Other prominent interhabitat differences in abundance were found in the Hymenoptera, which were comparatively more abundant in the teak plantation and Lepidoptera, which showed high abundance in the riverine habitat (Fig. 6).

The similarity between the three habitats in the proportionate abundance of different insect groups was calculated using the following formula[21]:

**I = 1 – 0.5(3 *ai-bi*)**

where **I** is the proportionate similarity between the two habitats, **ai** is the proportion of total individuals in habitat **a** that belong to the order **i,** and **bi** is proportion of total individuals in habitat **b** that belong to the order **i.**

**TABLE 1**
**Seasonal Pattern in the Abundance of Insect Groups in Different Habitats**

| | | Seasons | | | | | | | |
|---|---|---|---|---|---|---|---|---|---|
| | | NE Monsoon | | Winter | | Summer | | SW Monsoon | |
| Habitats | Insect group | Mean | S.E | Mean | S.E | Mean | S.E | Mean | S.E |
| Moist deciduous | Coleoptera | 4.3 | .9 | 1.6 | .3 | 3.8 | 1.1 | 7.5 | .4 |
| | Diptera | 14.5 | 2.1 | 9.3 | 1.3 | 4.7 | 1.1 | 37.1 | 7.9 |
| | Hemiptera | 4.3 | .5 | 4.9 | .6 | 4.9 | 1.1 | 7.3 | 1.2 |
| | Hymenoptera | 9.1 | 1.1 | 7.3 | .6 | 4.2 | .6 | 12.4 | 1.3 |
| | Lepidoptera | 5.7 | 1.7 | 13.5 | 1.1 | 1.4 | .3 | 7.8 | 2.0 |
| | Orthoptera | 5.9 | 1.1 | 14.4 | 2.1 | 4.6 | .8 | 7.5 | .9 |
| | Miscellaneous | 6.9 | 1.8 | 7.3 | 1.0 | 4.2 | .7 | 2.9 | .5 |
| | Total Insects | 50.7 | 3.8 | 58.1 | 4.2 | 27.9 | 2.3 | 82.4 | 8.2 |
| Riverine | Coleoptera | 5.4 | 1.2 | 2.9 | .4 | 6.2 | 1.6 | 12.4 | 1.0 |
| | Diptera | 15.5 | 3.1 | 8.5 | 1.8 | 4.4 | .8 | 21.5 | 2.0 |
| | Hemiptera | 2.8 | .5 | 6.8 | .7 | 3.6 | .5 | 2.8 | .4 |
| | Hymenoptera | 4.1 | .5 | 9.1 | .7 | 6.7 | .6 | 5.7 | 1.0 |
| | Lepidoptera | 5.9 | 1.6 | 13.1 | 1.5 | 4.3 | .7 | 16.1 | 3.3 |
| | Orthoptera | 7.5 | 1.0 | 10.1 | .9 | 5.1 | .8 | 9.4 | 1.7 |
| | Miscellaneous | 4.4 | .5 | 7.6 | .8 | 3.3 | .9 | 3.3 | .6 |
| | Total Insects | 45.5 | 2.9 | 58.0 | 2.7 | 33.5 | 2.9 | 71.3 | 5.4 |
| Teak plantation | Coleoptera | 3.2 | .8 | 2.8 | .3 | 3.5 | .9 | 6.2 | .9 |
| | Diptera | 7.4 | 1.0 | 3.6 | .5 | 2.2 | .6 | 16.3 | 3.4 |
| | Hemiptera | 2.7 | .6 | 3.8 | .7 | 2.9 | .6 | 6.3 | 1.0 |
| | Hymenoptera | 6.5 | .5 | 6.4 | .6 | 5.4 | 1.0 | 5.9 | 1.0 |
| | Lepidoptera | 2.1 | .6 | 4.9 | 1.2 | 1.2 | .3 | 4.0 | 1.1 |
| | Orthoptera | 5.0 | .7 | 6.0 | .7 | 3.8 | .4 | 7.4 | 1.0 |
| | Miscellaneous | 5.5 | 1.3 | 5.0 | .6 | 3.0 | .8 | 2.3 | .4 |
| | Total Insects | 32.3 | 2.0 | 32.3 | 2.8 | 22.1 | 1.9 | 48.3 | 3.4 |

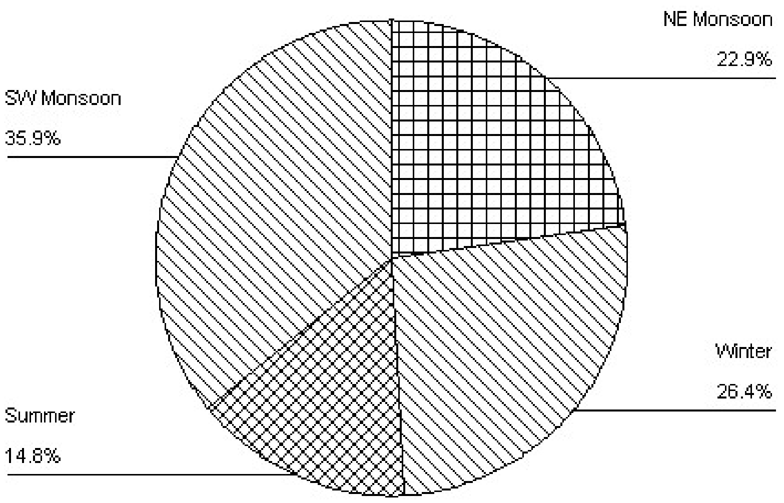

**FIGURE 4.** Overall seasonal abundance pattern of insects.

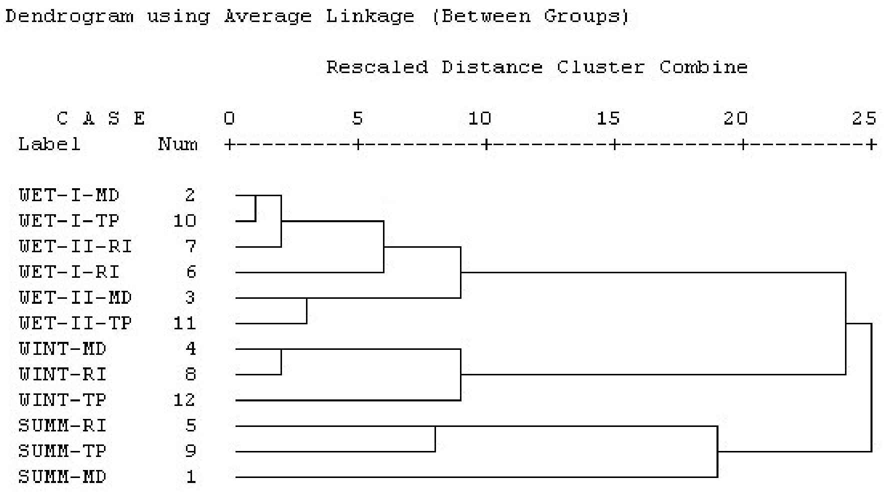

**FIGURE 5.** Dendrogram depicting the clustering based on community composition across habitats and seasons. SW= southwest; NE = northeast; MD = moist-deciduous; RI = riverine; TP = teak plantation.

**TABLE 2**
**Correlation Among the Insect Abundance in Different Habitats**

| | | Moist Deciduous | Riverine | Teak plantation |
|---|---|---|---|---|
| Pearson Correlation | Moist Deciduous | 1.000 | .676** | .907** |
| | Riverine | .676** | 1.000 | .727** |
| | Teak plantation | .907** | .727** | 1.000 |
| Sig. (2-tailed) | Moist Deciduous | . | .000 | .000 |
| | Riverine | .000 | . | .000 |
| | Teak plantation | .000 | .000 | . |
| N | Moist Deciduous | 24 | 24 | 24 |
| | Riverine | 24 | 24 | 24 |
| | Teak plantation | 24 | 24 | 24 |

**. Correlation is significant at the 0.01 level (2-tailed).

The indices obtained reveal a high degree of similarity among these three habitats. The moist-deciduous and riverine forests showed maximum similarity between them (I = 0.895), while the teak plantation habitat showed almost equal similarity indices with the moist-deciduous and riverine forests. However, the pattern of the temporal insect abundance fluctuation was more similar between the teak plantation and moist-deciduous habitats (r = 0.90663, $p < 0.001$).

## Species Richness

Precise identification of species in the samples is a major impediment in ecological studies involving arthropods, especially in the case of poorly studied groups such as forest insects. Classifying them into MTUs (or morpho-species) is one accepted means to overcome this taxonomic impediment in such ecological studies[22]. All the insects obtained in the sweeps were sorted out as if into different groups; it would have been done by an untrained eye, purely based on explicit morphological similarities. Further details on the pros and cons of this method of species segregation has been discussed by Derraik et al.[22]. The mean number of MTUs obtained per sample (100 sweeps) is considered as a measure of species richness in the present paper. The major drawback of using the MTUs was that the amount of species turnover between samples could not be accounted for.

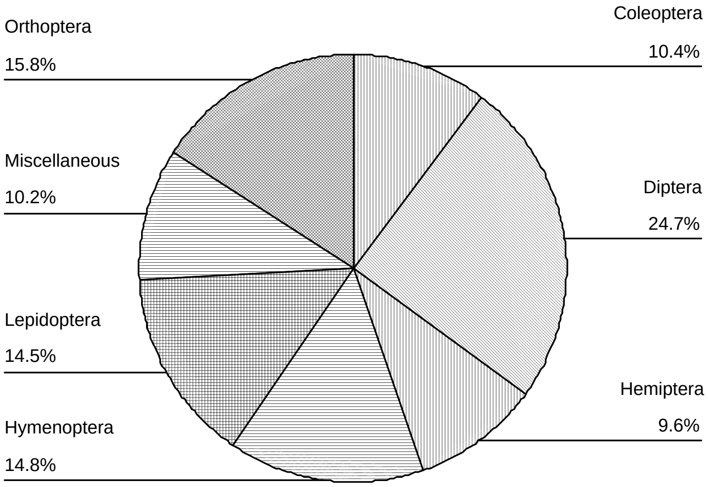

**FIGURE 6.** Overall composition of insect community.

The maximum species richness was recorded in the moist-deciduous (mean = 19.7 ± 0.85[SE] and Std. Deviation = 7.1) and riverine (mean = 19.6 ± 0.84[SE] and Std. Deviation = 7.0) forests. The teak plantation showed the least species richness in all the seasons (mean = 13.8 ± 0.5[SE] and Std. Deviation = 4.16). The average number of identifiable morpho-species obtained per 100 sweeps from different habitats in different seasons showed consistently low species richness in the teak plantation, in all the seasons compared to other two habitats (Fig. 7). Highest species richness was recorded in the moist-deciduous habitat during the rainy seasons (northeast and southwest monsoon seasons), while it was highest in the riverine habitat during winter and summer. The difference in the species richness of insects between the habitats might indicate the differences in plant diversity between the habitats.

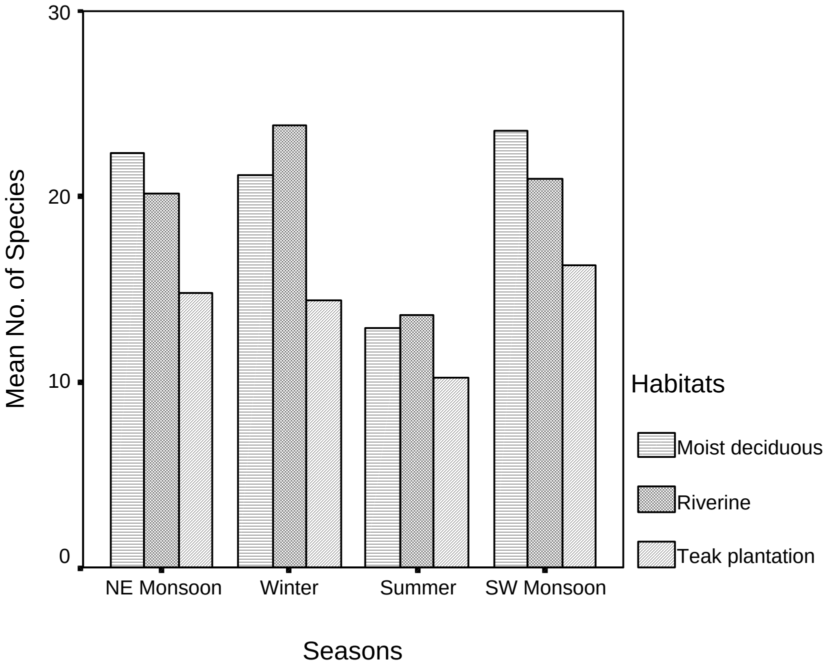

**FIGURE 7.** Mean species richness of insects.

## Role of Environmental Factors

An attempt was made to see the relationship between selected environmental parameters and the insect abundance. Five environmental parameters were monitored during the study, namely rainfall, number of rainy days, maximum temperature, minimum temperature, and relative humidity (Table 3). Among them, only the minimum temperature showed a significant negative correlation with the monthly fluctuations in insect abundance (r = –0.66; $p$ = 0.03). The total insect abundance had a significant cross-correlation with the rainfall at a lag of 3 months (r = 0.55, SE = 0.22). A multiple linear regression was derived using the environmental parameters (R = 0.766; Table 4)

**TABLE 3**
**Mean Monthly Values of Environmental Variables Monitored**

| | | Rainfall (mm) | No. of Rainy days | Humidity (%) | Max. temp. | Min. temp |
|---|---|---|---|---|---|---|
| Months | JANU | 8.5 | 2.5 | 60.0 | 34.0 | 24.0 |
| | FEBR | 5.0 | 1.0 | 40.0 | 36.0 | 25.0 |
| | MAR | 1.5 | 1.0 | 45.0 | 35.5 | 27.5 |
| | APRIL | 110.0 | 10.5 | 53.5 | 35.0 | 25.5 |
| | MAY | 100.5 | 8.5 | 54.5 | 37.0 | 26.5 |
| | JUNE | 409.0 | 16.5 | 82.0 | 32.5 | 24.5 |
| | JULY | 541.0 | 23.0 | 86.8 | 27.3 | 24.5 |
| | AUG | 157.5 | 15.0 | 78.3 | 30.7 | 23.5 |
| | SEPT | 280.5 | 11.5 | 72.5 | 31.5 | 24.0 |
| | OCT | 274.5 | 17.5 | 77.5 | 35.0 | 23.0 |
| | NOV | 201.5 | 13.5 | 77.3 | 31.7 | 22.7 |
| | DEC | 2.5 | 1.0 | 77.5 | 32.0 | 22.0 |

**TABLE 4**
**Regression Table for Insect Abundance and Environmental Variables**

**Coefficients[a]**

| Model | | Unstandardized Coefficients | | Standardized Coefficients | t | Sig. |
|---|---|---|---|---|---|---|
| | | B | Std. Error | Beta | | |
| 1 | (Constant) | 873.341 | 860.686 | | 1.015 | .330 |
| | Humidity | 5.593 | 3.757 | .550 | 1.489 | .162 |
| | Rainy days | 4.617 | 8.203 | .209 | .563 | .584 |
| | Rainfall | -.331 | .285 | -.394 | -1.162 | .268 |
| | Max. temp. | -14.685 | 16.753 | -.264 | -.877 | .398 |
| | Min. temp | -13.023 | 19.479 | -.148 | -.669 | .516 |

a. Dependent Variable: Insects

## CONCLUSION

Insect abundance in the understory vegetation varies significantly among the habitats. While natural habitats support a relatively high abundance of insects, the teak plantation supports much less. The comparison of the seasonal fluctuations in the insect abundance in different habitats of the mixed deciduous forest revealed that the seasonality of the overall insect population of the understory vegetation is controlled by the common macroclimatic factors rather than by any habitat-specific factors. The composition of the insect community in terms of proportions of different insect groups in the community also varies highly among seasons than among habitats. Although individually insignificant, abiotic environmental factors such as rainfall, number of rainy days, temperature, and humidity in combination showed a significant relation with insect abundance.

## ACKNOWLEDGEMENT

I gratefully acknowledge my senior colleague Dr. P. A. Azeez for all the encouragement and help provided. His valuable intellectual inputs through critical comments and fruitful discussions on the paper and during the manuscript preparation and subsequent revisions was crucial in shaping this paper into its present form. We gratefully acknowledge the kind help, cooperation and lively company provided by the field assistants Mr. Palani, Mr. Manoharan and the staff members of Tamilnadu Water Supply and Drainage Board (TWAD) and Forest Department at Siruvani forests, during the field investigations. Finally it is our pleasure to record our sincere thanks to the anonymous reviewer of the TheScientificWorldJournal, whose astute observations and constructive criticism on the initial manuscript of this paper helped us in improving the paper substantially.

## BIOSKETCH

**Dr. P.R. Arun** is a researcher specializing in ecological entomology, presently working with the Environmental Impact Assessment Division of Sálim Ali Centre for Ornithology and Natural History at Coimbatore in Southern India. Dr. Arun obtained his Ph.D. in 2001 from Bharathiar University of Tamilnadu for his thesis on seasonality and abundance of insects in a natural moist deciduous forest at Siruvani in Western Ghats, one of the biodiversity hotspots of the world. Current research interests include ecology of Papilionid butterfly communities of the Indian peninsula, indicator species, and environmental impact assessment.

**Dr. V S Vijayan** holds an M.Sc in Avian Biology from M.S. University, Baroda and Ph.D in field ornithology from the University of Bombay. 34 years of research career in field ornithology. Special interests include breeding strategies of birds and its relation to the seasonality of insects and phenology of plants. Currently Director of the Salim Ali Centre for Ornithology and Natural History.
